# A narrative review on the lactic acidosis and hypoglycemia in burkitt lymphoma: navigating metabolic changes

**DOI:** 10.1097/MS9.0000000000003819

**Published:** 2025-09-03

**Authors:** Rida Noor, Saad Khan, Tirath Patel, Mubashir Hussain, Mahnoor Asghar Keen, Shehram Tabassum, Lina Barman, Faraz Azhar, Mehrosh Khan, Rabbi Ali, Shumaila Butt, Richard M. Millis

**Affiliations:** a Department of Pathology, Faisalabad Medical University, Faisalabad Medical University, Pakistan; b Department of Medicine, Saidu Medical College, Swat, Pakistan; c Department of Medicine, Trinity Medical Sciences University School of Medicine, Ratho Mill Kingstown, Saint Vincent and the Grenadines; d Department of Medicine, Independent Medical College, Faisalabad, Pakistan; e Department of Medicine, Khyber Medical College, Peshawar, Pakistan; f Department of Medicine, Faisalabad Medical University, Faisalabad, Pakistan; g Department of Medicine, All India Institute of Medical Sciences, New Delhi, India; h Department of Medicine, Allama Iqbal Medical College, Lahore, Pakistan; i Department of Medicine, King Edward Medical University, Lahore Pakistan; j Department of Medicine, Sheikh Zayed Hospital, Rahim Yar Khan, Pakistan; k Department of Medicine, Jinnah Sindh Medical University, Karachi, Pakistan; l Department of Pathophysiology, American University of Antigua, Antigua and Barbuda

## Abstract

**Background:**

Burkitt lymphoma is a highly aggressive B-cell non-Hodgkin lymphoma closely associated with Epstein-Barr virus (EBV) infection and driven by MYC oncogene overexpression. It presents in three clinical forms: endemic, sporadic, and immunodeficiency-associated. Endemic Burkitt lymphoma, prevalent in Africa, typically involves jaw tumors; sporadic cases commonly present with abdominal masses; and immunodeficiency-associated cases occur in patients with HIV or other forms of immune compromise. A major clinical challenge is the development of severe metabolic complications, including lactic acidosis and hypoglycemia, resulting from rapid tumor proliferation and increased metabolic demands, particularly through enhanced glycolysis (the Warburg effect). These complications are often resistant to conventional supportive therapies and become life-threatening without urgent intervention.

**Purpose:**

To emphasize the clinical significance of early recognition and management of metabolic complications in Burkitt lymphoma and to examine the role of chemotherapy, particularly rituximab-based regimens, in improving patient outcomes.

**Methods:**

We conducted a comprehensive literature search across PubMed, Cochrane Library, and Google Scholar databases through September 2024 using keywords related to rituximab complications in Burkitt lymphoma. Studies were screened according to predefined criteria, focusing on lactic acidosis and hypoglycemia. Only English-language clinical trials, randomized controlled trials, and reviews were included. We excluded articles that did not include Burkitt lymphoma patients or address associated metabolic challenges and treatment-related toxicities.

**Conclusion:**

Early diagnosis and prompt chemotherapy initiation are critical for managing tumor burden and associated metabolic derangements in Burkitt lymphoma. While rituximab has significantly improved treatment outcomes, it must be used judiciously to minimize severe adverse effects. Timely intervention is particularly crucial in immunocompromised patients, where disease progression and treatment toxicity may be more severe.

## Introduction

Burkitt lymphoma is an aggressive subtype of non-Hodgkin B-cell lymphoma, characterized by the rapid proliferation of B-cells due to a translocation involving the MYC gene on chromosome 8^[1]^. There are three main subtypes: endemic, sporadic, and immunodeficiency-associated. Endemic Burkitt lymphoma is predominantly found in children from Central Africa, where it accounts for 30–50% of childhood cancers. This form typically presents with jaw lesions and is strongly associated with Epstein-Barr virus (EBV) infection and malaria^[2]^. Sporadic Burkitt lymphoma is more prevalent in developed regions such as North America and Europe, where it commonly presents with abdominal masses and accounts for 1–2% of adult lymphomas in these areas^[3]^.

The immunodeficiency-associated form is most frequently seen in individuals with HIV, where it is also linked to EBV in up to 30–40% of cases^[2]^. Clinical presentations can vary, but systemic “B” symptoms such as fever, night sweats, and weight loss are common. Abdominal symptoms, including pain, distension, nausea, and vomiting, are also frequently observed. Less commonly, Burkitt lymphoma presents with neurological symptoms, isolated nerve palsies, or masses in the ovaries or perineum, which can complicate diagnosis and treatment^[4,5]^.

Burkitt lymphoma primarily affects children, particularly those aged 3–9 years, but is also seen among the elderly and HIV-positive individuals with weakened immune systems^[1]^. The disease is especially prevalent in Central African countries, including Tanzania, Uganda, and Kenya, where it represents a significant proportion of pediatric cancers^[6]^. A history of malaria infection is strongly associated with an increased risk of developing Burkitt lymphoma in these regions^[7]^. Additionally, certain ethnic groups, such as Hispanics in the United States, are at a higher risk of developing the disease compared to other populations^[8]^.

The pathogenesis of Burkitt lymphoma is closely linked to the translocation of the c-MYC gene. c-MYC is a transcription factor that regulates B-cell proliferation. When the c-MYC gene translocates from chromosome 8 to chromosome 14, it results in uncontrolled B-cell growth, driving the development of Burkitt lymphoma^[9]^. Epstein-Barr virus (EBV) is another critical factor in the disease’s pathogenesis. EBV can facilitate the translocation of the c-MYC gene in B-cells, leading to the overexpression of complement receptor 2 (CR2) on B-cells, which EBV uses for infection, creating a self-perpetuating cycle^[10]^. Additionally, mutations in genes such as TP53 and TCF3 further contribute to disease progression and complexity.

The Warburg effect, characterized by increased aerobic glycolysis, is commonly observed in Burkitt lymphoma and results in elevated lactic acid production and metabolic acidosis. This metabolic alteration can significantly worsen a patient’s prognosis. In a study by Chaba et al., 34% of patients with aggressive Burkitt lymphoma demonstrated the Warburg effect, leading to a 70% one-year mortality rate^[11]^. This finding highlights the increased risk and poor prognosis associated with the Warburg effect in Burkitt lymphoma patients. The Warburg effect not only contributes to metabolic acidosis but also accelerates the production of nucleotides, which are used by malignant cells to sustain rapid neoplastic proliferation, perpetuating a cycle of aggressive tumor growth and metabolic disturbance^[12]^. This cycle exacerbates disease progression, resulting in poor clinical outcomes.

A hallmark of Burkitt lymphoma is the “starry sky” appearance, characterized by a background of densely packed lymphocytes interspersed with macrophages containing apoptotic debris from dying lymphocytes. This distinct histological pattern is typically associated with aggressive forms of the disease, which exhibit high tumor cell proliferation rates and are linked to lower survival rates^[13]^. Such morphological features underscore the aggressive nature of Burkitt lymphoma and its propensity for rapid progression. The presence of this histological characteristic can serve as a crucial indicator for clinicians in diagnosing and determining the prognosis for affected patients.

## Methodological framework and data collection techniques

Search strategy and data sources:

A comprehensive systematic literature search was conducted across multiple electronic databases including PubMed/MEDLINE, Cochrane Library, and Google Scholar from database inception through September 2024. The search strategy employed a combination of Medical Subject Headings (MeSH) terms and free-text keywords related to Burkitt lymphoma, rituximab, metabolic complications, lactic acidosis, and hypoglycemia. The specific search terms included: “Burkitt lymphoma,” “rituximab,” “lactic acidosis,” “hypoglycemia,” “metabolic complications,” “Warburg effect,” “tumor lysis syndrome,” “B-cell lymphoma,” and “chemotherapy complications.” Boolean operators (AND, OR) were used to combine search terms and optimize retrieval of relevant studies.

**HIGHLIGHTS**

- **Aggressive Nature of Burkitt Lymphoma:** Burkitt lymphoma is a highly aggressive B-cell malignancy associated with Epstein-Barr virus (EBV), characterized by rapid tumor cell proliferation and significant metabolic complications.
- **Metabolic Complications:** The rapid growth of tumor cells leads to severe metabolic issues, particularly lactic acidosis, and hypoglycemia, driven by the Warburg effect, which increases lactate production and depletes glucose.
- **Rituximab’s Dual Role:** While Rituximab improves treatment outcomes by targeting CD20 on B-cells, it also increases the risk of infections and other complications, necessitating careful patient management.
- **Prognostic Implications:** The presence of lactic acidosis is linked to poor prognosis, indicating severe metabolic derangements that can lead to multi-organ dysfunction and increased mortality risk.
- **Challenges in Diagnosis:** Symptoms of lactic acidosis and hypoglycemia often overlap with general cancer-related symptoms, complicating timely diagnosis and intervention.
- **Importance of Early Treatment:** Prompt initiation of chemotherapy is crucial for addressing both the underlying malignancy and the associated metabolic disturbances, improving survival rates.
- **Emerging Research Directions:** Ongoing studies focus on targeted therapies aimed at the MYC oncogene and improved risk stratification to enhance treatment efficacy for patients with Burkitt lymphoma.
- **Rituximab-associated Complications:** Awareness of potential complications from Rituximab therapy, including infections and inflammatory bowel diseases, is essential for optimizing patient care during treatment.

Inclusion criteria:

- Studies reporting on patients diagnosed with burkitt lymphoma according to who classification criteria.
- Studies investigating rituximab-based treatment regimens and associated complications
- Articles focusing on metabolic complications, specifically lactic acidosis and/or hypoglycemia
- Clinical trials, randomized controlled trials (RCTs), observational studies, case series, and systematic reviews
- Studies published in English language
- Studies involving both pediatric and adult populations
- Articles reporting on treatment-related toxicities and metabolic disturbances

Exclusion Criteria:

- Studies not including Burkitt lymphoma as part of the study population
- Articles that did not address metabolic challenges or treatment-related toxicities
- Case reports with fewer than adequate clinical detail
- Studies focusing exclusively on other lymphoma subtypes without Burkitt lymphoma patients
- Non-English language publications
- Letters to editors, editorials, and conference abstracts without full-text availability
- Studies with insufficient data on clinical outcomes or metabolic parameters

## Discussion

### Epidemiology of burkitt lymphoma

Burkitt lymphoma (BL) in East Africa, particularly in malaria-endemic regions of Uganda, Tanzania, and Kenya, is strongly linked to chronic Plasmodium falciparum malaria and Epstein-Barr virus (EBV) infections. Chronic malaria suppresses T-cell immunity, enabling EBV reactivation and B-cell proliferation, which are key to BL development. The disease mainly affects children aged 5–9 years, with a male-to-female ratio of 2–3:1, and is associated with rural residence, poor sanitation, and limited healthcare access. Nutritional deficiencies, especially protein-energy malnutrition, may further increase susceptibility^[6]^. BL occurs in three main types with distinct epidemiological patterns. The endemic form, common in equatorial Africa and Papua New Guinea, affects mainly children aged 4–7 years, with an incidence of 4–6 per 100,000, and is almost always EBV-positive. The sporadic form, found in North America and Europe, is rare (0.2–0.3 per 100,000), affects older children and adults, and shows lower EBV association (10–20%). The immunodeficiency-associated type, seen in HIV or transplant patients, has a higher incidence (~22 per 100,000), affects adults around 40–45 years, and shows moderate EBV involvement (25–40%). These variations highlight the influence of geography, immunity, and viral factors in BL epidemiology.Table 1 summarizes key epidemiologic features across BL subtypes^[7]^.

**Table 1:** Epidemiology of Burkitt Lymphoma

Parameter	Details
Global Incidence	Approximately 3-5% of all non-Hodgkin lymphoma worldwide
High-Incidence Regions	Endemic in sub-Saharan Africa and New Guinea
Endemic Burkitt lymphoma	Affects children aged 4-7 years; associated with EBV in 95% cases
Sporadic Burkitt lymphoma	Common in western countries; accounts for 1-2% adult lymphomas
Immunodeficiency associated	Common in HIV AIDS patients; linked to EBV infection in 30-40% of cases
Age Group	Bimodal peaks in children and adults over 60 years
Gender Distribution	Male to Female ratio of 2:1
Ethnic Predisposition	Higher incidence in African and Afro-Caribbean population
Etiology	Strongly associated with EBV, Malaria and immune dysfunction
Prognosis	Higher cure rates with prompt and intensive chemotherapy^[7]^

### Role of epstein-barr virus proteins in burkitt lymphoma oncogenesis

Epstein-Barr virus (EBV) proteins, including LMP-1 and EBNA-1, play crucial roles in the oncogenic transformation of B-cells in Burkitt lymphoma. LMP-1 (Latent Membrane Protein 1) functions as a constitutively active receptor that mimics CD40 signaling, thereby promoting B-cell survival and proliferation^[14]^. This protein activates key oncogenic pathways, including NF-κB, PI3K/AKT, and JAK/STAT, which foster cell growth and confer resistance to apoptosis. EBNA-1 (Epstein-Barr Nuclear Antigen 1) is essential for maintaining EBV episomes during cell division and indirectly contributes to genomic instability and oncogenesis by disrupting cellular DNA repair processes^[15]^.

### Impact of HIV and immunodeficiency on epstein-barr virus-driven burkitt lymphoma

Immunodeficiency, particularly that caused by HIV infection, increases the risk of developing Burkitt lymphoma by impairing the immune system’s ability to control Epstein-Barr virus (EBV) infection. In healthy individuals, the immune system, particularly EBV-specific cytotoxic T cells, maintains effective control over EBV-infected B cells. However, in HIV-positive individuals, immune surveillance is compromised due to reduced CD4 + T cell counts, allowing unchecked EBV replication and B-cell transformation, which increases the likelihood of oncogenesis and Burkitt lymphoma development^[16]^.

### Mechanisms of apoptosis evasion and microenvironment adaptation in burkitt lymphoma

Burkitt lymphoma cells evade apoptosis primarily through deregulation of key signaling pathways that normally promote cell death, thereby contributing to their pathogenicity. One of the principal mechanisms involves overexpression of the MYC oncogene, which drives unchecked cell proliferation. Although MYC can induce apoptosis under normal conditions, Burkitt lymphoma cells counteract this effect by upregulating anti-apoptotic proteins such as BCL-2 or through mutations that inactivate pro-apoptotic proteins like p53. These alterations disrupt the intrinsic apoptotic pathway, allowing cancer cells to survive despite genetic damage or stress, ultimately promoting tumor growth and treatment resistance^[17]^.

The tumor microenvironment further facilitates apoptosis evasion in Burkitt lymphoma. Stromal cells, including follicular dendritic cells and macrophages, secrete cytokines and chemokines such as IL-10, TNF-α, and CXCL12, which enhance lymphoma cell survival and proliferation. These signals activate critical pathways, including NF-κB and JAK/STAT, thereby providing additional support for tumor growth. Additionally, the hypoxic microenvironment induces metabolic adaptations that enable Burkitt lymphoma cells to thrive under nutrient-limited conditions^[18]^.

### Distinctive features of burkitt lymphoma: genetic, clinical, and biological insights

Burkitt lymphoma is a highly aggressive and distinct subtype of non-Hodgkin lymphoma (NHL), recognized for its unique genetic, clinical, and biological characteristics that differentiate it from other NHL types. The distinctions between Burkitt lymphoma and other forms of NHL span several domains, including genetic alterations, growth patterns, clinical presentation, association with Epstein-Barr virus (EBV), epidemiology, and response to therapy^[19]^. One of the defining features of Burkitt lymphoma is the presence of a characteristic chromosomal translocation involving the MYC oncogene on chromosome 8. The most common translocation, t(8;14), places the MYC gene under the control of the immunoglobulin heavy chain (IGH) locus, resulting in its overexpression^[20]^.

### Advancements in targeted therapies and immunotherapy for burkitt lymphoma

Recent advancements in Burkitt lymphoma research and treatment have focused on improving patient outcomes through targeted therapies, immunotherapies, and enhanced risk stratification. A major area of progress involves the development of targeted therapies directed at the MYC oncogene, which drives the aggressive growth characteristic of Burkitt lymphoma. Researchers are investigating MYC pathway inhibitors as well as drugs targeting associated signaling pathways, including PI3K/AKT and mTOR. Furthermore, novel combinations of chemotherapy with monoclonal antibodies, particularly rituximab (which targets the CD20 protein on B-cells), have significantly improved survival rates^[21]^.

### Challenges and management of lactic acidosis and hypoglycemia in burkitt lymphoma

Diagnosing lactic acidosis and hypoglycemia in patients with Burkitt lymphoma is particularly challenging due to the aggressive nature of the disease and the complex metabolic disturbances it induces. The rapid proliferation of Burkitt lymphoma cells leads to increased glycolysis, which contributes to the development of lactic acidosis, while the high metabolic demands of both the tumor and its treatment can result in hypoglycemia. Symptoms such as fatigue and confusion often overlap with general cancer-related symptoms or side effects of chemotherapy, making timely diagnosis more difficult^[22]^. Additionally, these metabolic complications can develop rapidly, sometimes even before a definitive diagnosis of Burkitt lymphoma is established, further complicating early identification.

Prompt initiation of chemotherapy is crucial, as it significantly improves outcomes for Burkitt lymphoma patients experiencing lactic acidosis and hypoglycemia by addressing the underlying cause of these metabolic disturbances rather than merely managing the symptoms. The disease is characterized by rapid cell turnover and heightened metabolic activity, which can lead to tumor lysis syndrome—a condition that further increases the risk of lactic acidosis and hypoglycemia. While alkali therapy may provide temporary correction of acidosis, it does not address the malignancy itself, allowing the disease to progress and potentially worsening metabolic derangements^[23]^.

### Impact of lactic acidosis on prognosis in burkitt lymphoma

The relationship between Burkitt lymphoma, lactic acidosis, and patient prognosis is both significant and complex. Burkitt lymphoma is a highly aggressive B-cell malignancy characterized by rapid cell proliferation and high metabolic demand, often resulting in metabolic complications such as lactic acidosis. This condition arises when tumor cells undergo accelerated glycolysis, producing excess lactate, particularly in the context of tumor lysis syndrome. The presence of lactic acidosis in patients with Burkitt lymphoma is associated with a poor prognosis, as it reflects severe metabolic derangements that can lead to multi-organ dysfunction and an increased risk of mortality^[24]^.

### Diagnostic challenges of lactic acidosis in burkitt lymphoma patients

Lactic acidosis typically presents with non-specific symptoms such as fatigue, weakness, nausea, vomiting, and confusion. These symptoms overlap considerably with common manifestations of Burkitt lymphoma, including general malaise, fever, and chemotherapy-related side effects. The rapid progression of Burkitt lymphoma can further exacerbate these symptoms, making it challenging for clinicians to differentiate between cancer-related fatigue and underlying metabolic disturbances^[25]^.

### Key indicators for investigating burkitt lymphoma in patients with metabolic disturbances

Key indicators for investigating Burkitt lymphoma in patients presenting with unexplained lactic acidosis and hypoglycemia include patient age and demographic factors, particularly in children aged 3–9 years and immunocompromised individuals such as those with HIV. Systemic symptoms—such as fever, night sweats, and significant weight loss, collectively known as “B symptoms”—may signal an underlying malignancy^[26]^. Rapid clinical deterioration, notable laboratory findings such as elevated lactate levels and persistent hypoglycemia, and imaging results revealing masses in the abdomen or central nervous system further support the need for prompt investigation. Together, these factors underscore the importance of thorough evaluation when metabolic abnormalities are present, as they may indicate aggressive forms of Burkitt lymphoma^[27]^.

### Impact of lactic acidosis on mortality in burkitt lymphoma patients

Lactic acidosis significantly increases mortality rates in patients with Burkitt lymphoma due to its association with aggressive tumor growth and critical metabolic disturbances. Elevated lactate levels result from the rapid proliferation of malignant B-cells, leading to enhanced anaerobic glycolysis. This metabolic state not only signals a poor prognosis but is also linked to severe complications, including respiratory failure and cardiovascular collapse. Literature indicates that mortality rates for Burkitt lymphoma patients experiencing lactic acidosis can exceed 70%, often occurring within weeks of diagnosis. Therefore, prompt recognition and initiation of treatment are essential for improving survival outcomes^[28]^.

### Mechanism of action of rituximab in treating burkitt lymphoma

**Figure 1.**
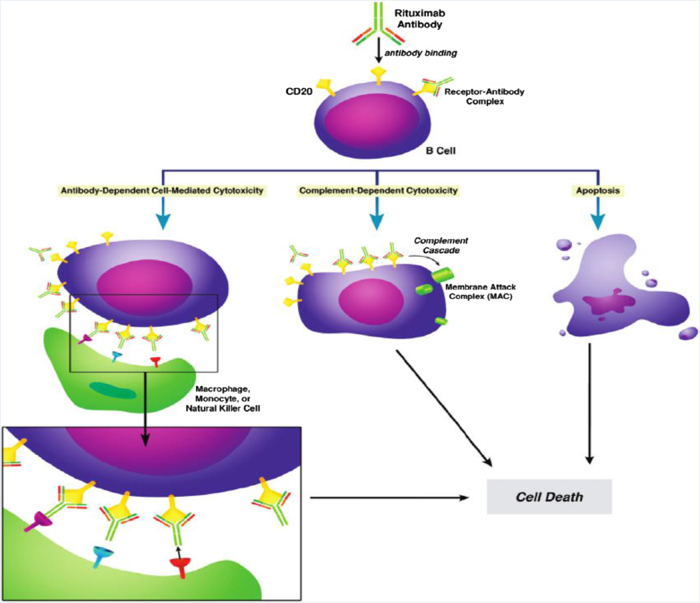
Shows the mechanism of action of rituximab^[29]^.

Rituximab is a chimeric monoclonal antibody that targets the CD20 antigen on the surface of B-cells, leading to their depletion through several key mechanisms. Upon binding to CD20, rituximab initiates complement-dependent cytotoxicity (CDC), activating the complement cascade and resulting in the formation of membrane attack complexes that lyse B-cells. Additionally, it engages immune effector cells via Fc gamma receptors to mediate antibody-dependent cellular cytotoxicity (ADCC), recruiting natural killer cells, macrophages, and other immune cells to eliminate the targeted B-cells. Rituximab also directly induces apoptosis in B-cells by disrupting survival signaling pathways. These mechanisms collectively contribute to its efficacy in treating B-cell lymphomas and autoimmune diseases^[28,29]^. The mechanism of action of rituximab is summarized in Figure 1.

### Rituximab-induced crohn’s disease: a case report and implications for gut health

The use of rituximab has led to significant improvements in treating hematological malignancies and autoimmune diseases. Although rituximab-associated ulcerative colitis has been previously documented, we present two novel cases of rituximab-induced Crohn’s disease in elderly patients undergoing lymphoma treatment. Both patients exhibited inflammation, ulceration, and granulomas consistent with Crohn’s disease, and showed positive responses to immunosuppressive therapy. This association between rituximab and ileocolitis suggests that CD20 + lymphocytes might play a protective role in the gut, and their depletion could contribute to the onset or worsening of inflammatory bowel diseases^[30]^.

### Strategies to minimize rituximab-associated complications in burkitt lymphoma treatment

To minimize the risk of rituximab-associated complications, several evidence-based strategies should be implemented. Pre-treatment screening for infections, particularly hepatitis B, is essential, as rituximab can reactivate dormant viral infections. For patients with prior hepatitis B exposure, antiviral prophylaxis is recommended throughout the treatment course. Premedication with corticosteroids and antihistamines effectively reduces the risk of infusion-related reactions, which commonly occur during the initial infusion^[31]^. Regular monitoring of immunoglobulin levels during treatment helps prevent prolonged immunosuppression, and immunoglobulin replacement therapy may be necessary for patients experiencing recurrent infections due to hypogammaglobulinemia. Dose modifications and reduced infusion rates can further minimize severe adverse reactions in high-risk patients. Vigilant monitoring for signs of progressive multifocal leukoencephalopathy (PML), though rare but potentially fatal, should be conducted through routine neurological assessments^[32]^.

Despite its efficacy in treating various B-cell malignancies and autoimmune diseases, rituximab has demonstrated limited effectiveness in certain conditions, particularly chronic lymphocytic leukemia (CLL). In CLL, rituximab monotherapy has shown modest response rates, especially in patients with high-risk disease characteristics or those who have received multiple prior treatment regimens. While studies demonstrate that combining rituximab with chemotherapy improves clinical outcomes, its use as a single agent is insufficient to achieve durable responses in most CLL patients^[33]^.

### Rare but serious risks associated with rituximab treatment in burkitt lymphoma

Rituximab, while effective against B-cell malignancies, carries rare but serious risks requiring close monitoring. These include **progressive multifocal leukoencephalopathy (PML)** due to JC virus reactivation, **severe mucocutaneous reactions** like Stevens-Johnson syndrome and toxic epidermal necrolysis, and **tumor lysis syndrome (TLS)** leading to life-threatening metabolic disturbances. **Hepatitis B reactivation** can result in liver failure, especially in previously exposed individuals. **Cardiovascular events** may occur, particularly in those with underlying heart conditions. Additionally, **prolonged hypogammaglobulinemia** heightens infection risk. These complications necessitate vigilant surveillance and early intervention during rituximab therapy^[34]^

### Mortality rate associated with rituximab

The 2023 retrospective study by Alhamadh et al. examined 137 adult patients treated with rituximab for hematologic and autoimmune conditions. Rituximab dosing ranged from 375 mg/m^2^ to 1,000 mg per infusion, with an average cumulative dose of ~ 3.2 g. The overall mortality was 22.6%, primarily from severe infections. Hypogammaglobulinemia occurred in 43.8%. Notably, recipients of the 1,000 mg dose had lower infection and mortality risks, while those on 375–500 mg dosing saw more frequent hypogammaglobulinemia and higher mortality. Key mortality risk factors included corticosteroid use, hematological malignancy, hypogammaglobulinemia, and ICU admission, underscoring the need for close monitoring around RTX therapy. This study provides a clear estimate: **rituximab treatment was associated with a 22.6% mortality** in the cohort, primarily driven by infections, and higher dosing (1,000 mg) appeared **protective** compared to standard dosing. Table 2 presents the consolidated view of mortality, infection risk, and hypogammaglobulinemia by clinical subgroup and dosing.^[35]^

**Table 2:** Mortality Rates, Infection Risks, and Hypogammaglobulinemia Incidence Among Adult Patients Treated with Rituximab: Impact of Dosing Regimens

Patient Group	RTX Dose	Mortality Rate	Comments
Overall	Mixed 375-1000 mg)	22.6%	Mostly infection- related
Age <30 years	Mostly 375 gm	0%	No deaths observed
Age 31-50 years	Mixed	33.3%	Higher than younger group
Age >51 years	Mixed	66.7%	Highest age related mortality
Diabetes	Mixed	55.6%	Major comorbidity risk
Hypertension	Mixed	33.3%	Elevated risk
CKD	Mixed	33.3%	Increased risk
Steroid Use (yes)	Mixed	88.9%	Major mortality driver
Steroid Use (no)	Mixed	11.1%	Much more lower risk
Hypoglobulinemia (yes)	Mixed	55.6%	Higher mortality
Hypoglobulinemia (no)	Mixed	44.4%	Lower mortality
ICU admission (yes)	Mixed	66.7%	High risk
ICU admission (no)	Mixed	11.5%	Lower risk
RTX 1000 mg	1000 mg	—	Associated with lower mortality and infection risk
RTX	375-500 mg	—	Higher hypoglobulinemia and mortality^[35]^

CKD: Chronic Kidney Disease; RTX: Rituximab.

### Challenges in rituximab treatment: cost, accessibility, and vaccination strategies

The use of rituximab, an effective treatment for various hematological malignancies and autoimmune diseases, presents challenges related to cost, accessibility, and vaccination strategies. Rituximab is often expensive, with treatment cycles frequently exceeding $10,000, which can limit access, particularly in regions with limited healthcare resources^[36]^. Vaccination is critical for these patients to prevent infections; however, immunosuppression resulting from rituximab therapy can reduce vaccine efficacy. Ideally, vaccinations should be administered prior to initiating rituximab treatment. Live vaccines are contraindicated, while inactivated vaccines may be given, although their effectiveness may be diminished. As a result, careful timing and planning are essential to optimize the protective benefits of vaccinations for patients receiving rituximab therapy^[37]^.

### Impact of lactic acidosis on burkitt lymphoma: incidence and mortality in pediatric patients

Lactic acidosis is a recognized complication in patients with Burkitt lymphoma, most often resulting from rapid tumor cell turnover and increased glycolytic activity. While the exact incidence can vary, a study published in the Irish Medical Journal reported that among 33 pediatric patients with Burkitt lymphoma, one patient (approximately 3%) died during induction therapy due to refractory lactic acidosis^[38]^. This finding is consistent with other case reports and reviews, which highlight that lactic acidosis—particularly type B lactic acidosis—is a rare but life-threatening metabolic complication in Burkitt lymphoma. The development of lactic acidosis in these patients is closely linked to the Warburg effect, where malignant cells preferentially utilize aerobic glycolysis, leading to excessive lactate production even in the presence of adequate oxygenation. Importantly, several case studies have shown that lactic acid levels typically decrease only after the initiation of effective combination chemotherapy, underscoring the need for rapid diagnosis and prompt oncologic treatment to improve outcomes. Overall, the presence of lactic acidosis in Burkitt lymphoma is an indicator of poor prognosis and requires urgent, targeted intervention.

### Association between lactic acidosis and survival in adult burkitt lymphoma patients: the role of LDH levels

Lactic acidosis is a recognized complication in patients with Burkitt lymphoma, typically stemming from increased glycolytic activity. In a study published in the *American Journal of Medicine*, researchers followed 28 adults with Burkitt lymphoma and found that those whose baseline serum lactate dehydrogenase (LDH) level exceeded 500 U/L had significantly shorter survival than patients with lower LDH levels^[39]^.

### Mortality and prognostic factors of lactic acidosis in non-hodgkin lymphoma

Furthermore, a comprehensive literature review indicates that lactic acidosis induced by non-Hodgkin lymphoma is associated with a 73% mortality rate within the first month and a 92% overall mortality rate. Clinical outcomes are closely linked to the tumor’s responsiveness to chemotherapy^[40]^.

### Incidence and case reports of lactic acidosis in burkitt lymphoma patients

Lactic acidosis is a recognized but rare complication in patients with Burkitt lymphoma. While the exact number of reported cases is not well documented in the literature, several case reports have highlighted this association. For example, one report described a 60-year-old man who developed lactic acidosis related to Burkitt lymphoma^[41]^, while another detailed a 67-year-old man presenting with severe lactic acidosis in the context of the disease^[42]^. Additionally, a case involving a 39-year-old male with type B lactic acidosis secondary to newly diagnosed Burkitt lymphoma has been documented^[43]^. These cases underscore the importance of recognizing lactic acidosis as a potential, albeit uncommon, manifestation of Burkitt lymphoma.

### Statistical data showing prevalence of lactic acidosis & hypoglycemia in burkitt lymphoma

Burkitt lymphoma (BL) is a highly aggressive B-cell non-Hodgkin lymphoma characterized by rapid tumor proliferation and a propensity for central nervous system (CNS) involvement. Metabolic complications, including lactic acidosis and hypoglycemia, are significant concerns in BL patients. Roberts et al. reported that BL patients often present with lactic acidosis, a condition resulting from increased lactate production due to the Warburg effect, where tumor cells favor glycolysis over oxidative phosphorylation. This metabolic shift leads to elevated lactate levels and acidosis, contributing to patient morbidity and mortality.^[44]^ Sharma et al highlighted that CNS involvement in BL is associated with poor prognosis and increased mortality. Their study emphasized the need for vigilant monitoring and early intervention to manage neurological symptoms effectively.^[45]^ Smith et al. discussed the occurrence of hypoglycemia in BL patients, attributing it to the high metabolic demands of rapidly proliferating tumor cells consuming large amounts of glucose. Untreated hypoglycemia can lead to neurological symptoms and increased mortality, underscoring the importance of prompt recognition and management^[46]^.

### Treatment of lactic acidosis in burkitt lymphoma: approaches and therapeutic strategies

Effective management of type B lactic acidosis in hematologic malignancies—such as Burkitt lymphoma—relies on swiftly addressing the underlying tumor while providing metabolic support. Early initiation of chemotherapy significantly reduces lactate levels by curbing tumor glycolysis. Meanwhile, supportive measures like intravenous sodium bicarbonate infusions help correct severe acidosis, and renal replacement therapy may be required in cases of fluid overload or kidney impairment. Ahmed & Bansal (2019) emphasize that delayed chemotherapy often results in persistently elevated lactate levels and worsened morbidity, highlighting the crucial interplay between oncologic control and metabolic stabilization. Table 3 summarizes the practical bedside steps for managing lactic acidosis and hypoglycemia^[47]^.

**Table 3:** Treatment strategy

Condition	Treatment Strategy	Details
Lactic acidosis	Discontinue chemotherapy that may worsen acidosis	Rituximab, methotrexate,and other chemotherapy agents can precipitate acidosis
	Correct underlying metabolic disturbances (hyperkalemia, hypoglycemia)	Metabolic disturbances must be corrected to prevent acidosis.
	IV Sodium Bicarbonate (if pH<7.1)	To buffer the acid and raise the pH to prevent further complications.
	Hemodialysis or continuous renal replacement therapy	In case of severe acidosis or renal failure, these methods can help clear excess lactate and correct acid-base balance
	Aggressive hydration with IV fluids	To improve renal perfusion and facilitate lactate clearance
	Monitor lactate levels regularly	Monitoring lactate helps guide therapy
Hypoglycemia	IV glucose administration	Immediate treatment to increase blood glucose to safe level
	Oral glucose	In patients who can take oral food
	Octreotide(if associated with Insulin secretion)	For patients with insulin like growth factor, octreotide can suppress insulin secretion
	Careful monitoring of blood glucose	To guide further therapy and adjust glucose administration^[47]^

### Metabolic complications in burkitt lymphoma: a case of lactic acidosis and hypoglycemia

Khanal et al. reported a case of severe lactic acidosis and hypoglycemia in a patient with Burkitt lymphoma, attributed to the Warburg effect—a phenomenon in which tumor cells preferentially utilize glycolysis for energy production even in the presence of oxygen. This metabolic shift leads to excessive lactate accumulation and glucose depletion. Prompt recognition and early intervention with glucose, bicarbonate, and chemotherapy were critical in stabilizing the patient’s condition. The case underscores the importance of identifying metabolic disturbances as potential paraneoplastic manifestations in aggressive malignancies such as Burkitt lymphoma^[48]^.

### Relapsed mantle cell lymphoma presenting with lactic acidosis and hypoglycemia

A 67-year-old man with relapsed mantle cell lymphoma developed severe lactic acidosis and hypoglycemia. Although his metabolic abnormalities normalized following chemotherapy and intensive care, he ultimately succumbed to ventilator-associated pneumonia. This case highlights the urgent need for early recognition and management of metabolic emergencies in patients with lymphoma^[49]^.

### Spontaneous tumor lysis syndrome in thoracic burkitt lymphoma

A 20-year-old woman presented with a large mediastinal mass causing lactic acidosis and spontaneous tumor lysis syndrome. Despite aggressive management, including chemotherapy, the patient developed multi-organ failure and died. This case underscores the aggressive nature of thoracic Burkitt lymphoma and the potential for rapid metabolic deterioration^[50]^.

### Atypical presentation of burkitt lymphoma with isolated peritoneal involvement

A 39-year-old male exhibited type B lactic acidosis and recurrent hypoglycemia, secondary to newly diagnosed Burkitt lymphoma with isolated peritoneal involvement. The case emphasizes the importance of considering malignancy in unexplained lactic acidosis and hypoglycemia, even with atypical presentations^[51]^.

### Primary breast burkitt lymphoma with lactic acidosis in a child

A 16-year-old female presented with bilateral breast swelling and respiratory distress. She developed severe lactic acidosis, which only improved after initiating combination chemotherapy. Despite partial remission, the patient succumbed to fungal septicemia. This case highlights the rarity of primary breast involvement in pediatric Burkitt lymphoma and the prognostic significance of lactic acidosis^[52]^.

### Clinical presentation of warburg effect in aggressive lymphoma

A 76-year-old woman with double-expressor diffuse large B-cell lymphoma presented with severe lactic acidosis and extreme hypoglycemia. Initial management included bicarbonate infusion and chemotherapy, but complications like tumor lysis syndrome and liver failure led to her demise. The case illustrates the poor prognosis associated wiType B Lactic Acidosis in High-Grade Hematologic Malignancies th the Warburg effect in aggressive lymphomas^[53]^.

### Tumor Lysis Syndrome (TLS) and lactic acidosis

Tumor lysis syndrome (TLS) results from rapid tumor cell breakdown, leading to electrolyte imbalances such as hyperkalemia, hyperuricemia, hyperphosphatemia, and hypocalcemia. Prophylactic measures typically include aggressive hydration, allopurinol, and rasburicase. In contrast, lactic acidosis arises from increased anaerobic metabolism, often due to tissue hypoxia. In one case of Burkitt lymphoma, TLS prophylaxis with rasburicase and hydration was implemented; however, the patient subsequently developed lactic acidosis following aggressive chemotherapy. Both conditions necessitate vigilant monitoring and prompt management to prevent life-threatening organ failure^[54]^.

### Type B lactic acidosis in high-grade hematologic Malignancies

Type B lactic acidosis is a rare but serious complication of hematologic malignancies such as Burkitt lymphoma, occurring in the absence of tissue hypoperfusion. It results from tumor-induced metabolic reprogramming, notably the Warburg effect, wherein rapidly proliferating tumor cells preferentially utilize glycolysis over oxidative phosphorylation—even in oxygen-rich conditions—leading to excessive lactate production. Furthermore, hepatic infiltration or dysfunction can impair lactate clearance, exacerbating acidosis. Elevated lactate dehydrogenase (LDH) levels serve as a surrogate marker of high tumor burden and are associated with poor prognosis. In one reported case, a 38-year-old man with Burkitt lymphoma developed severe lactic acidosis (lactate 12.4 mmol/L, LDH >3,000 U/L) without hypotension, which resolved following the prompt initiation of chemotherapy^[55]^.

### Immunophenotyping in burkitt lymphoma

Burkitt lymphoma typically expresses CD10, CD19, CD20, and BCL6, with a Ki-67 proliferation index approaching 100%, reflecting its highly proliferative nature. These immunophenotypic markers help differentiate it from other aggressive lymphomas, such as diffuse large B-cell lymphoma (DLBCL), which often shows a lower Ki-67 index and variable expression of BCL6 and BCL2. Confirmation of a MYC gene translocation via fluorescence in situ hybridization (FISH) further supports the diagnosis. Immunophenotyping is therefore crucial for accurate classification and therapeutic decision-making^[56]^.

### Diagnostic workup for hematologic malignancies

**Figure 2.**
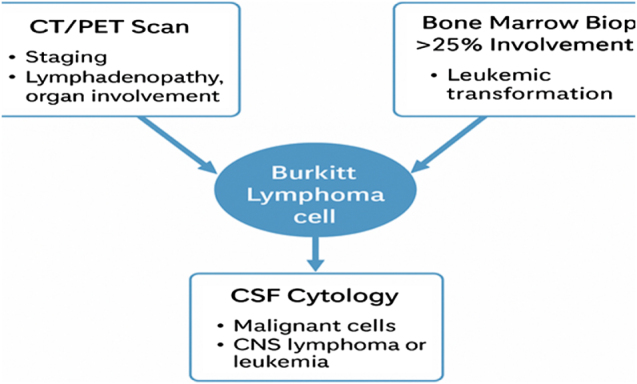
Illustrates diagnostic work up of Burkitt lymphoma^[57]^.

Imaging with CT/PET scans is essential for staging, identifying lymphadenopathy, organ involvement, and assessing metabolic activity. A bone marrow biopsy revealing >25% involvement is indicative of leukemic transformation, a critical diagnostic marker. In cases of suspected CNS involvement, CSF cytology can detect malignant cells, guiding treatment for CNS lymphoma or leukemia. Early detection of bone marrow and CNS infiltration is crucial for prognosis and therapeutic planning. These diagnostic tools ensure comprehensive assessment, optimizing patient management^[57]^. An example diagnostic work-up pathway is depicted in Figure 2.

### Rituximab timing, CNS prophylaxis, and prognostic considerations in burkitt lymphoma

In Burkitt lymphoma, the addition of rituximab to regimens like R-EPOCH has significantly improved outcomes, but its potent cytotoxic effect can trigger tumor lysis syndrome (TLS) in patients with high tumor burden; hence, delayed rituximab administration (e.g., after initial cytoreduction) is sometimes employed to minimize risk. Due to the high risk of central nervous system (CNS) relapse, especially in cases with bone marrow or testicular involvement, intrathecal methotrexate or cytarabine is routinely used as CNS prophylaxis^[58]^. Regimens like CODOX-M/IVAC and dose-adjusted R-EPOCH have demonstrated overall survival rates up to 90% in low-risk patients. A case involving a 39-year-old male with high LDH and marrow involvement showed successful management with delayed rituximab, intrathecal methotrexate, and CODOX-M/IVAC, leading to sustained remission^[59]^.

### Prognostic factors in burkitt lymphoma

Prognosis in Burkitt lymphoma is influenced by several factors, with poor outcomes associated with elevated LDH, CNS or bone marrow involvement, bulky tumor burden, and poor performance status. While early, aggressive treatment often results in high cure rates, prognosis worsens significantly in the presence of metabolic complications such as lactic acidosis or tumor lysis syndrome^[60]^. In one case, a 42-year-old male presented with abdominal distension, fatigue, and confusion. Workup showed a Ki-67 index of 100%, LDH >3,500 U/L, bone marrow involvement, and elevated lactate (10.2 mmol/L). Despite initiation of intensive chemotherapy, he developed refractory lactic acidosis and succumbed within days, highlighting the grave prognosis associated with advanced disease and metabolic instability^[61]^.

### Unique teaching points: lactic acidosis in burkitt lymphoma

Lactic acidosis is a rare but serious paraneoplastic syndrome observed in aggressive malignancies like Burkitt lymphoma due to rapid cell turnover and metabolic reprogramming (the Warburg effect). Unlike more indolent lymphomas such as follicular lymphoma or mantle cell lymphoma, which typically do not cause metabolic derangements, Burkitt lymphoma can present with severe lactic acidosis. This case emphasizes the importance of understanding oncogenic metabolism in real-world clinical care, particularly in high-grade lymphomas^[62]^. A 33-year-old male with Burkitt lymphoma presented with fatigue, confusion, and lactate levels of 12.3 mmol/L, alongside elevated LDH (4,000 U/L). Despite aggressive chemotherapy, he developed multi-organ failure due to worsening acidosis, underlining the grave implications of metabolic dysfunction in rapidly proliferating tumors^[55]^.

### MYC overexpression, warburg effect, and lactic acidosis in burkitt lymphoma

MYC overexpression is a hallmark of Burkitt lymphoma and drives tumor growth through dysregulation of cellular metabolism. The Warburg effect, a shift to aerobic glycolysis, is a key metabolic adaptation in MYC-driven cancers, where cells increase glucose uptake and conversion to lactate even in the presence of oxygen. This metabolic reprogramming supports rapid cell proliferation but results in lactic acidosis, as the increased lactate production exceeds the cell’s ability to clear it. In Burkitt lymphoma, this effect is particularly pronounced due to the high turnover rate of tumor cells, which overwhelms both liver and renal clearance mechanisms, leading to lactic acidosis^[62]^. A case report of a 29-year-old male with Burkitt lymphoma presented with abdominal pain, confusion, and elevated lactate (12.4 mmol/L) alongside LDH >3,000 U/L. Despite aggressive chemotherapy, including rituximab, the patient developed refractory lactic acidosis, linked to rapid tumor cell turnover and MYC-driven metabolic reprogramming. The patient unfortunately succumbed to multi-organ failure secondary to acidosis, underscoring the severity of metabolic dysfunction in MYC-overexpressing lymphomas^[63]^.

### Future research directions in managing lactic acidosis and hypoglycemia in burkitt lymphoma

Future research on lactic acidosis and hypoglycemia in Burkitt lymphoma should focus on elucidating the tumor’s metabolic activity and rapid cell turnover. Investigating the underlying mechanisms of its glycolytic phenotype may identify novel therapeutic targets to improve prognosis. Additionally, the discovery of early biomarkers for metabolic complications could enable timely intervention. Developing effective management strategies for tumor lysis syndrome and related metabolic derangements is critical. Personalized treatment approaches based on individual metabolic profiles should be prioritized. Collaboration among oncologists, endocrinologists, and metabolic specialists will be essential to advance care. Such multidisciplinary efforts have the potential to improve patient outcomes and reduce mortality.

## Conclusion

Lactic acidosis and hypoglycemia are critical metabolic complications in Burkitt lymphoma, driven by the aggressive proliferation of malignant B-cells that enhance glycolysis, leading to elevated lactic acid and depleted glucose levels. This metabolic reprogramming, known as the Warburg effect, is associated with a high mortality rate—studies show that approximately 73% of patients with Burkitt lymphoma–related lactic acidosis experience poor outcomes, often within one month of diagnosis. Although rituximab has improved survival in Burkitt lymphoma, it can increase the risk of infections and potentially worsen metabolic disturbances. Therefore, a comprehensive management approach targeting both the malignancy and its metabolic complications is essential to improve patient outcomes. Future research should focus on optimizing treatment protocols and developing supportive strategies to reduce these risks and enhance quality of life.

## Data Availability

Data is publicly available.

## Research registration unique identifying number (UIN)

Research registration unique identifying number was not required for the narrative review.
